# Phosphorus Application Enhances Root Traits, Root Exudation, Phosphorus Use Efficiency, and Seed Yield of Soybean Genotypes

**DOI:** 10.3390/plants12051110

**Published:** 2023-03-01

**Authors:** Mohammad Salim, Yinglong Chen, Zakaria M. Solaiman, Kadambot H. M. Siddique

**Affiliations:** 1 The UWA Institute of Agriculture, and UWA School of Agriculture and Environment, The University of Western Australia, Perth, WA 6009, Australia; 2 Bangladesh Agricultural Research Institute, Gazipur 1701, Bangladesh

**Keywords:** soybean genotypes, contrasting root systems, root morphology, carboxylates, phosphorus use efficiency, seed yield

## Abstract

Phosphorus (P) is a vital macronutrient required for soybean growth and development but is a finite resource in agriculture worldwide. Low inorganic P availability in soil is often a significant constraint for soybean production. However, little is known about the response of P supply on agronomic, root morphology, and physiological mechanisms of contrasting soybean genotypes at various growth stages and the possible effects of different P on soybean yield and yield components. Therefore, we conducted two concurrent experiments using the soil-filled pots with six genotypes (deep-root system: PI 647960, PI 398595, PI 561271, PI 654356; and shallow-root system: PI 595362, PI 597387) and two P levels [0 (P0) and 60 (P60) mg P kg^−1^ dry soil] and deep PVC columns with two genotypes (PI 561271 and PI 595362) and three P levels [0 (P0), 60 (P60), and 120 (P120) mg P kg^−1^ dry soil] in a temperature-controlled glasshouse. The genotype × P level interaction showed that increased higher P supply increased leaf area, shoot and root dry weights, total root length, shoot, root, and seed P concentrations and contents, P use efficiency (PUE), root exudation, and seed yield at different growth stages in both experiments. At the vegetative stage (Experiment 1), shallow-rooted genotypes with shorter life cycles had more root dry weight (39%) and total root length (38%) than deep-rooted genotypes with longer life cycles under different P levels. Genotype PI 654356 produced significantly higher (22% more) total carboxylates than PI 647960 and PI 597387 under P60 but not at P0. Total carboxylates positively correlated with root dry weight, total root length, shoot and root P contents, and physiological PUE. The deep-rooted genotypes (PI 398595, PI 647960, PI 654356, and PI 561271) had the highest PUE and root P contents. In Experiment 2, at the flowering stage, genotype PI 561271 had the greatest leaf area (202%), shoot dry weight (113%), root dry weight (143%), and root length (83%) relative to the short-duration, shallow-rooted genotype PI 595362 with external P applied (P60 and P120), with similar trends at maturity. PI 595362 had a greater proportion of carboxylates as malonate (248%), malate (58%), and total carboxylates (82%) than PI 561271 under P60 and P120 but no differences at P0. At maturity, the deep-rooted genotype PI 561271 had greater shoot, root, and seed P contents and PUE than the shallow-rooted genotype PI 595362 under increased P rates but no differences at P0. Further, the genotype PI 561271 had higher shoot (53%), root (165%), and seed yield (47%) than PI 595362 with P60 and P120 than P0. Therefore, inorganic P application enhances plant resistance to the soil P pool and maintains high soybean biomass production and seed yield.

## 1. Introduction

Soybean (*Glycine max*) has become a vital agricultural crop, with a steady increase in global annual production due to its excellent nutritional and health benefits [1]. It is cultivated worldwide, often in low-phosphorus (P) soil due to intensive erosion, weathering, and strong P fixation by free Fe and Al oxides [2]. Therefore, low P availability is often a significant constraint and is critical to improving soybean production [3]. Simultaneously, the world is facing a future shortage of P resources as P fertilizers rapidly increase in cost. It has been estimated that rock phosphate, a P source, will be exhausted by the end of this century [4]. External P is used widely to enhance soybean production, but its use is associated with several global environmental challenges [5].

Soybeans generally need more P like other legume species because N_2_-fixing root nodules are strong P sinks [6,7]. Plant roots can cope with low P availability by developing several morpho-physiological changes within plants [8], including (i) extending the root system to explore soil, increasing root P acquisition, and (ii) intensifying root exudation to mobilize P in the rhizosphere to make soil P more available [9]. It is also evident that P uptake differs genetically between soybean cultivars. P-efficient soybean cultivars often efficiently acquire soil P to improve soybean production [3]. Rhizosphere carboxylates formed acidification, which could be an essential strategy to increase P availability and plant P uptake [10,11]. By decreasing Fe^3+^ acidification, dissolving phosphate complexes, and increasing net P uptake, plants maintain growth in P-deficient soils, demonstrating that root physiological traits may be more important than root morphological traits for P use efficiency (PUE) in P-impoverished soils [12]. Few studies have shown low-P resistance at later growth stages when P-deficiency symptoms are evident [13,14,15]. However, there is limited information on the agronomic, root morphology, and physiological mechanisms of contrasting soybean genotypes at different growth stages in response to P supply, different P responses on soybean yield, and yield components. We conducted two concurrent experiments using soil-filled pots (six genotypes, Experiment 1) and a 1.0 m-deep PVC column (two genotypes, Experiment 2) in a glasshouse. We hypothesized that (i) different P levels affect root growth, PUE, and root exudation at different growth stages; (ii) significant variation in yield and yield components exist among soybean genotypes with contrasting root systems in response to P levels, and (iii) contrasting soybean genotypes develop different adaptation strategies in response to P supply.

## 2. Results

### 2.1. Experiment 1

#### 2.1.1. Shoot and Root Traits: Harvested 49 DAS (Days after Sowing) at the Vegetative Stage

**Figure 1:**
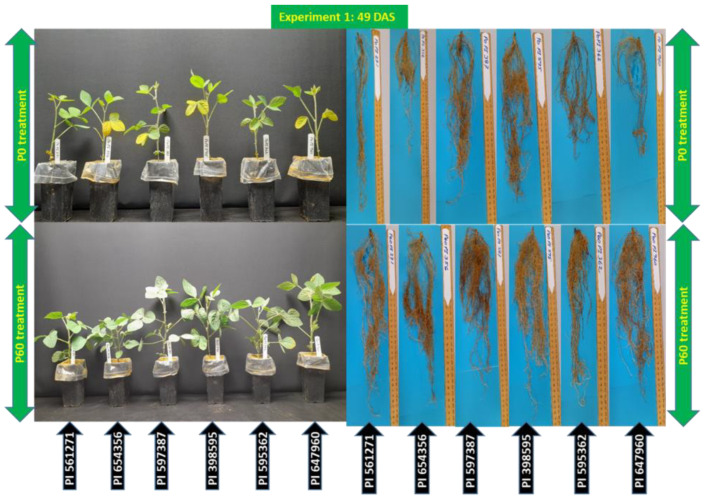
Shoot and root growth of six soybean genotypes grown in rectangular pots (Expt. 1). The plants were harvested 49 days after sowing (DAS) under 0 (P0) and 60 (P60) mg kg^−1^ dry soil.

Plant height, leaf area, shoot and root-related traits, and SPAD value were all affected by the genotype × P interaction (*p* < 0.05, Table 1 and Supplementary Table S1). PI 597387, PI 647960, PI 398595, and PI 595362 had taller plants than PI 654356 and PI 561271, with taller plants under P60 than P0 (*p* < 0.05, Figure 1, Supplementary Table S1). PI 398595 and PI 561271 had greater (60% more) leaf areas under P60. PI 398595 had the most shoot dry weight at P60, with the other five genotypes producing less but similar shoot dry weights; all tested genotypes had similar shoot dry weights under P0 (*p* < 0.05, Figure 1, Table 1). On average, PI 597387, PI 647960, PI 398595, and PI 561271 produced 39% more root dry weight than PI 654356 and PI 595362 under P60, a much greater difference than under P0. PI 597387 and PI 398595 had the most root dry weight under P0 (*p* < 0.05, Figure 1, Table 1). PI 597387, PI 647960, and PI 398,595 produced significantly more total dry weight than the other genotypes under P60 (*p* < 0.05, Table 1). PI 597387 and PI 398595 had higher root:shoot ratios than PI 561271, under P0 than P60 (*p* < 0.05, Table 1). The genotype × P interaction affected total root length; PI 597387 produced more root length (38% higher) than the other genotypes, while PI 654356 produced the least under P60 and P0 (*p* < 0.05, Figure 1, Table 1). Average root diameter and rhizosphere pH did not have significant genotype × P interactions but had significant genotype and P effects, respectively (Table 1 and Supplementary Table S1). Rhizosphere pH decreased with increasing P rate (Supplementary Table S1). The average root diameter increased with increasing P dose at P60 than P0. PI 398595 had the thinnest roots than other genotypes under both P levels (Table 1). For significant P effects, P60 produced significantly greater plant height, leaf area, shoot dry weight, root dry weight, total dry weight, total root length, root:shoot ratio, SPAD value, and root length density than P0, while the reverse was true for root:shoot ratio and rhizosphere pH (Figure 1, Table 1, Supplementary Table S1).

#### 2.1.2. Carboxylates Measured in the Rhizosphere Roots at the Vegetative Stage

**Figure 2:**
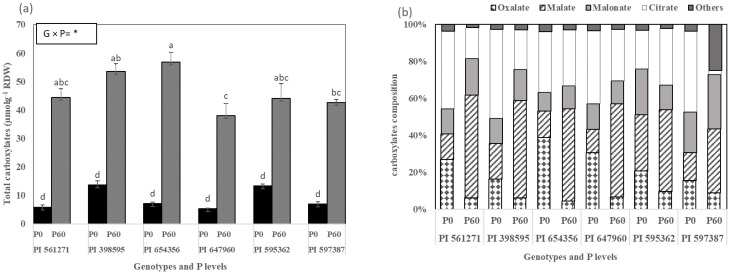
(**a**) Total carboxylates and (**b**) carboxylate composition of six soybean genotypes grown in rectangular pots (Expt. 1). The plants were harvested 49 days after sowing (DAS) under 0 (P0) and 60 (P60) mg kg*−1* dry soil. Values are the mean of four replicates (2 plants pot^−1^). Mean data of total carboxylates followed by different letters differ significantly at *p* < 0.05 using Turkey’s test. Vertical error bars indicate standard error of the mean (s.e.m, *n* = 4). ANOVA: * significant difference at *p* < 0.05. RWD = root dry weight, G × P = Genotype phosphorus interaction.

The total amount of rhizosphere carboxylates—citrate, malonate, malate, oxalate, and others (trace amount detected: acetate, lactate, succinate, fumarate, cis-aconitate, and shikimate)—based on root dry weight had a significant genotype × P interaction

(*p* < 0.05, Figure 2a,b). PI 398595, PI 561271, PI 595362, and PI 654356 produced similar and significantly higher (22%) total carboxylates than PI 647960 and PI 597387 under P60 (*p* < 0.05, Figure 2a). Total carboxylates under P0 did not significantly differ between genotypes. However, PI 398595 and PI 595362 secreted more organic acids than the other four genotypes (Figure 2a). PI 595362 and PI 597387 had the highest (15 % more) oxalate amount under P60 (Figure 2b). PI 398595, PI 561271, PI 595362, and PI 654356 secreted a similar amount of malate, significantly higher (34%) than PI 647960 and PI 597387 under P60. PI 597387 secreted significantly higher (172%) malonate than PI 647960, and other genotypes secreted a similar amount of malonate, different from PI 647960 under P60 (Figure 2b). PI 654356 secreted significantly higher (1742%) citrate than PI 597387 under P60. PI 597387 secreted a significantly greater (27%) total amount of other organic acids (acetate, lactate, succinate, fumarate, cis-aconitate, shikimate) under P60 than the other genotypes at both P levels (Figure 2b). All genotypes increased carboxylate exudation under P60 relative to P0, except for oxalate and citrate, which decreased in PI 654356 and PI 597387 (Figure 2b).

#### 2.1.3. Shoot and Root P Acquisition and PUE at the Vegetative Stage

**Figure 3:**
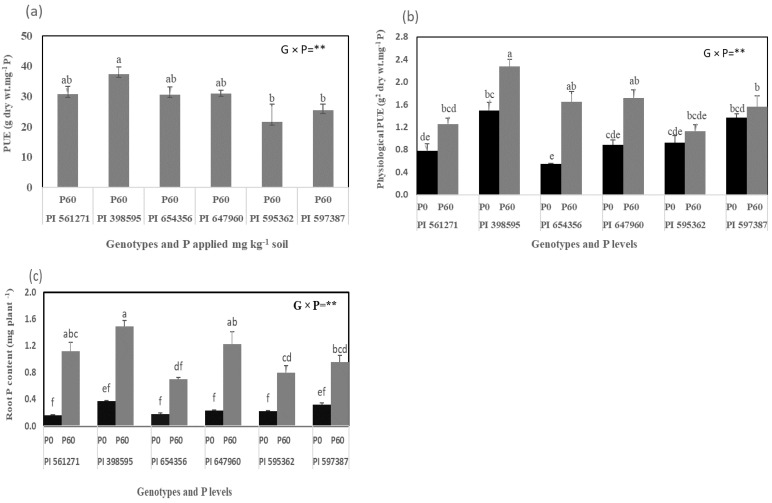
(**a**) Phosphorus use efficiency (PUE), (**b**) physiological PUE, and (**c**) root P content of six soybean genotypes grown in the rectangular pots (Expt. 1). The plants were harvested 49 days after sowing (DAS) under 0 (P0) and 60 (P60) mg kg^−1^ dry soil. Values are the mean of four replicates (2 plants pot^−1^). Mean data of each trait followed by different letters are significantly different at *p* < 0.05 using Turkey’s Test. Vertical error bars represent s.e.m (*n* = 4). ANOVA: ** significant difference at *p* < 0.01. PUE = Phosphorus (P) use efficiency, G × P = Genotype phosphorus interaction.

Root P content, agronomic PUE, and physiological PUE had significant genotype × P interactions (*p* < 0.01, Figure 3a–c). P60 produced significantly higher root P contents and physiological PUE than P0 for all genotypes (Figure 3b,c). In particular, PI 398595, PI 647960, and PI 561271 had, on average, 54% greater root P contents than the other three genotypes under P60 but similar root P contents under P0 (Figure 3c). PI 597387, PI 561271, and PI 595362 had equal and lower physiological PUE than the other three genotypes under P60 (Figure 3b). PI 398595, PI 647960, PI 654356, and PI 561271 had similar and higher agronomic PUE than PI 597387 and PI 595362 under P60 (*p* < 0.01, Figure 3a). P60 significantly increased shoot P concentration, shoot P content, and root P concentration relative to P0 (*p* < 0.001, Supplementary Table S1), but there were no genotype or genotype × P effects.

#### 2.1.4. Pearson’s Correlation and Principal Component Analysis (PCA) among Different Traits at the Vegetative Stage

Shoot dry weight positively correlated with root dry weight, total root length, oxalic acid, malic acid, malonic, citric acid, total carboxylates, shoot P content, root P content, and physiological PUE (r = 0.90, 0.81, 0.55, 0.89, 0.82, 0.60, 0.91,0.93, 0.91, 0.77, respectively; *p* < 0.001, Table 2). Root dry weight positively correlated with total root length, oxalic acid, malic acid, malonic acid, citric acid, total carboxylates, shoot P content, root P content, and physiological PUE (r = 0.95, 0.49, 0.75, 0.79, 0.45, 0.79, 0.81, 0.88, 0.76, respectively; *p* < 0.001). Total root length positively correlated with oxalic acid, malic acid, malonic acid, total carboxylates, shoot P content, root P content, and physiological PUE (r = 0.53, 0.61, 0.79, 0.69, 0.74, 0.78, 0.68, respectively; *p* < 0.001, Table 2), and correlated with citric acid (r = 0.29; *p* < 0.05). Oxalic acid is positively associated with malic acid, malonic acid, total carboxylates, shoot P content, and root P content (r = 0.51, 0.62, 0.62, 0.60, 0.48, respectively; *p* < 0.001) and citric acid (r = 0.35; *p* < 0.01). Malic acid positively correlated with malonic acid, citric acid, total carboxylates, shoot P content, root P content, and physiological PUE (r = 0.76, 0.76, 0.97, 0.86, 0.84, 0.61, respectively; *p* < 0.001). Malonic acid positively correlated with total carboxylates, shoot P content, root P content, physiological PUE (r = 0.84, 0.82, 0.72, 0.54, respectively; *p* < 0.001), and citric acid (r = 0.33; *p* < 0.05, Table 2). Citric acid positively correlated with total carboxylates, shoot P content, root P content, and physiological PUE (r = 0.75, 0.52, 0.53, and 0.50, respectively; *p* < 0.001, Table 2). Total carboxylates positively correlated with shoot P content, root P content, and physiological PUE (r = 0.87, 0.83, 0.63, respectively; *p* < 0.001). Shoot P content positively correlated with root P content and physiological PUE (r = 0.88, and 0.51; *p* < 0.001). Root P content positively correlated with physiological PUE (r = 0.64; *p* < 0.001, Table 2).

**Figure 4:**
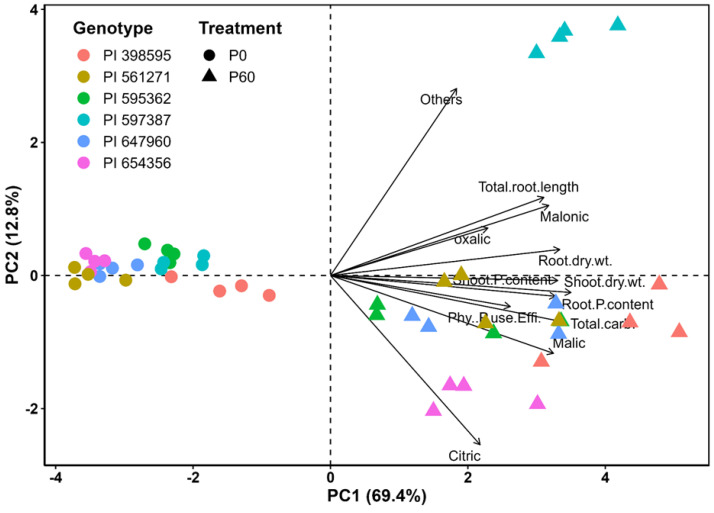
Principal component analysis matrix of root morphological and physiological traits in the soybean genotypes from Experiment 1. The plants were harvested 49 days after sowing (DAS) under 0 (P0) and 60 (P60) mg kg^−1^ dry soil.

Ten root morphological and physiological traits were included in the principal component analysis. Two principal components (PCs) were identified with eigenvalues >1, capturing 82.2% of the total variation in root system architectural and root physiological traits across the six soybean genotypes (Figure 4). The first component (PC1) represented 69.4% of the variability and accounted primarily root morphological and physiological traits such as shoot dry weight, root dry weight, total root length, oxalic acid, malic acid, malonic acid, citric acid, total carboxylates, shoot P contents, and root P contents with six genotypes and P60 supply. The second component (PC2) represented 12.8% of the variation derived from six soybean genotypes and P0 supply (Figure 4).

### 2.2. Experiment 2

#### 2.2.1. Shoot and Root Traits at the Flowering Stage

**Figure 5:**
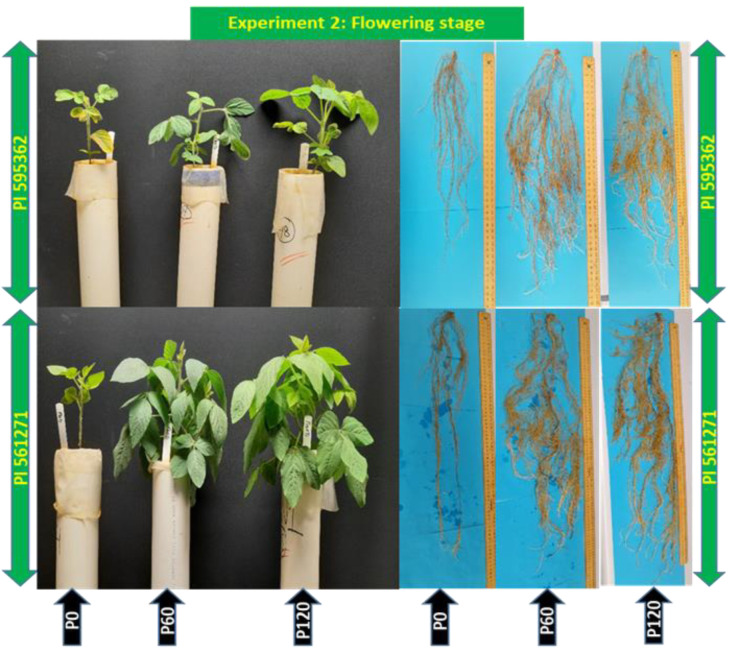
Shoot and root growth of two soybean genotypes grown in 1.0 m-deep PVC columns (Expt. 2). The plants were harvested at the flowering stage (appearance of first flower) at 49 DAS (PI 595362) and 67 DAS (PI 561271) under 0 (P0), 60 (P60), and 120 (P120) mg kg^−1^ dry soil.

The genotype × P interaction significantly affected days to flowering, plant height, leaf area, shoot dry weight, root dry weight, total dry weight, total root length, root length density, root surface area, root volume, and rhizosphere pH (*p* < 0.05, Table 3 and Supplementary Table S2). A significant genotype effect occurred for root:shoot ratio (*p* < 0.01), and significant P effects occurred for average root diameter and SPAD (*p* < 0.05, *p* < 0.001). PI 561271 had the highest leaf area (202%), shoot dry weight (113%), root dry weight (143%), and root length (83%) at P60 and P120 than P0 (Figure 5, Table 3, Supplementary Table S2). The root:shoot ratio of PI 561271 significantly differed from PI 595362 (Table 3). Average root diameter and SPAD value were higher under P60 and P120 than under P0. Rhizosphere pH decreased with P application (Table 3, Supplementary Table S2).

#### 2.2.2. Carboxylates Measured from Rhizosphere Root at the Flowering Stage

**Figure 6:**
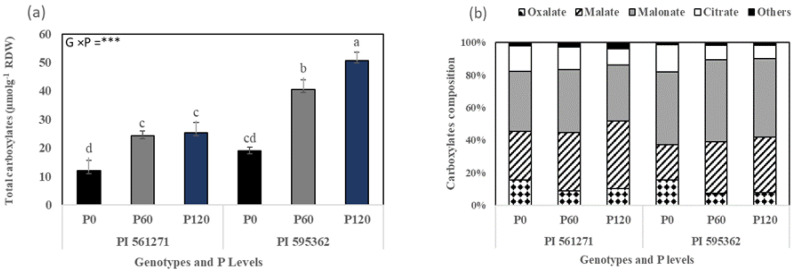
(**a**) Total carboxylates and (**b**) carboxylate composition of two soybean genotypes grown in 1.0 m-deep PVC columns (Expt. 2). The plants were harvested at the flowering stage (appearance of the first flower) at 49 DAS (PI 595362) and 62 DAS (PI 561271) under 0 (P0), 60 (P60), and 120 (P120) mg kg^−1^ dry soil. Values are mean of four replicates (1 plant column^−1^). Mean data for total carboxylates followed by different letters differ significantly at *p* < 0.05 using Turkey’s test. Vertical error bars represent s.e.m (*n* = 4). ANOVA: *** indicate significant difference at *p* < 0.001. RWD = root dry weight.

The genotype × P interaction significantly affected total rhizosphere carboxylates, citrate, malonate, and malate based on root dry weight (*p* < 0.05, Figure 6a,b). Significant P effects occurred for other carboxylates (acetate, lactate, succinate, fumarate, cis-aconitate, shikimate) and oxalate, but no genotype or genotype × P effects were observed. PI 595362 produced significantly higher (82%) total carboxylates than PI 561271 under P60 and P120 but no differences at P0 (*p* < 0.001, Figure 6a). At the same time, increasing the P application increased the amount of all carboxylates in both genotypes. However, total carboxylates in PI 561271 did not significantly differ between P60 and P120 (Figure 6a). Overall, the small-rooted PI 595362 released more carboxylates than the large-rooted PI 561271 under P60 and P120, while no difference was observed between genotypes under P0 (Figure 6a). PI 595362 had a greater proportion of carboxylates as malonate (248%) and malate (58%) than PI 561271 under P60 and P120 than P0 (Figure 6b *p* < 0.001).

#### 2.2.3. Shoot and Root P Acquisition and PUE at the Flowering Stage

**Figure 7:**
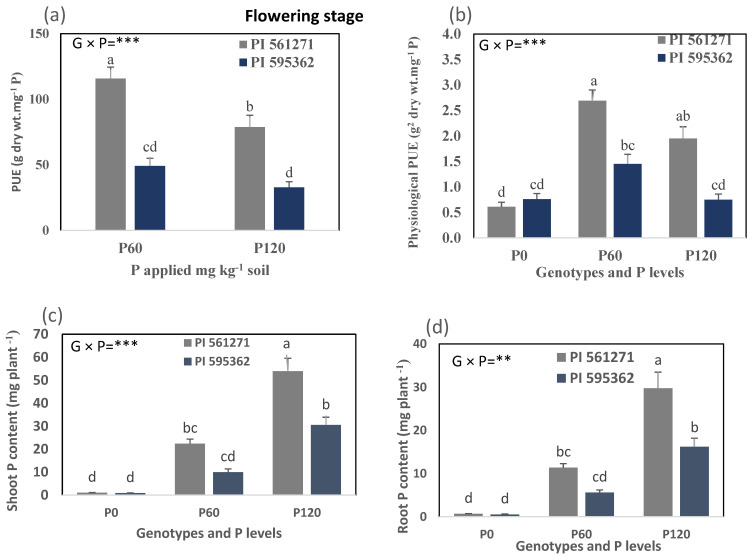
(**a**) PUE, (**b**) physiological PUE, (**c**) shoot P content, and (**d**) root P content of two soybean genotypes grown in the 1.0 m-deep PVC columns (Expt. 2). The plants were harvested at the flowering stage (appearance of the first flower appeared) at 49 DAS (PI 595362) and 67 DAS (PI 561271) under 0 (P0), 60 (P60), and 120 (P120) mg kg^−1^ dry soil. Values are means of four replicates (1 plant column^−1^). Mean data for each trait followed by different letters differ significantly t at *p* < 0.05 using Turkey’s test. Vertical error bars represent s.e.m (*n* = 4). ANOVA: ** and *** indicate significant differences at *p* < 0.01 and *p* < 0.001. PUE = phosphorus (P) use efficiency.

The genotype × P interaction significantly affected shoot and root P concentrations, shoot and root P contents, PUE, and physiological PUE (*p* < 0.05, Supplementary Table S2, Figure 7a–d). PI 595362 had higher shoot and root P concentrations than PI 561271 under P60 and P120 than under P0 (*p* < 0.05, Supplementary Table S2). In contrast, PI 561271 had higher shoot and root P contents than PI 595362 under P60 and P120 than under P0 (*p* < 0.01, Figure 7c,d). Under P120, PI 561271 had 76% more shoot P content and 83% more root P content than PI 595362. The genotype × P interaction significantly decreased PUE and physiological PUE under P60 and P120 relative to P0 in both genotypes (*p* < 0.001, Figure 7a,b). PI 561271 had significantly higher PUE and physiological PUE than PI 595362 under P60 and P120 than P0 (*p* < 0.001, Figure 7a,b). Phosphorus application significantly increased (262% more) shoot and root P concentrations and contents but decreased PUE. Physiological PUE increased in both genotypes under P60 and P120 relative to P0 at the start of flowering (Supplementary Table S2, Figure 7a–d).

#### 2.2.4. Shoot, Root, and Seed P Acquisition, PUE at Maturity

**Figure 8:**
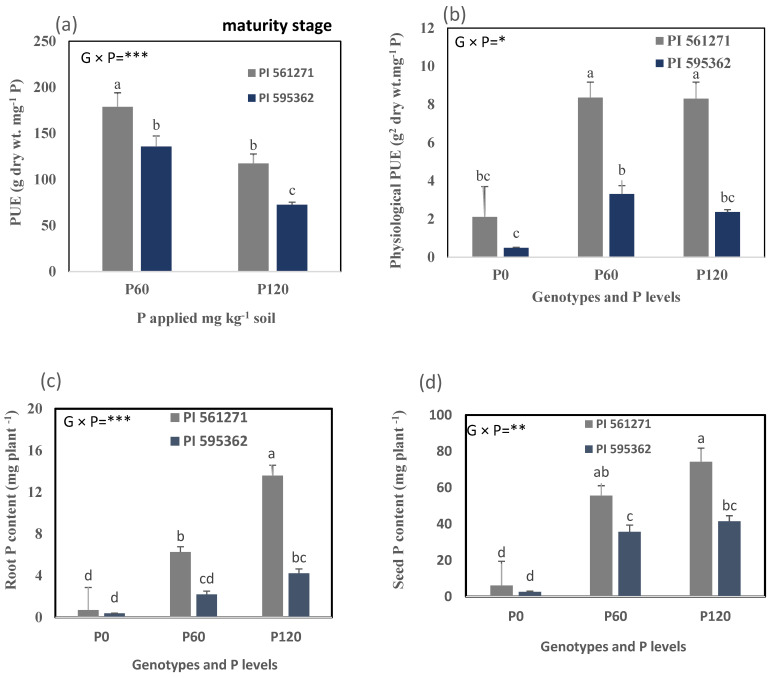
(**a**) PUE, (**b**) physiological PUE, (**c**) root P content, and (**d**) seed P content of two soybean genotypes grown in 1.0 m-deep PVC columns (Expt. 2). The plants were harvested at maturity stage at 105 DAS (PI 595362) and 143 DAS (PI 561271) under 0 (P0), 60 (P60), and 120 (P120) mg kg^−1^ dry soil. Values are means of four replicates (1 plant column^−1^). Mean data for each trait followed by different letters differ significantly at *p* < 0.05 using Turkey’s test. Vertical error bars represent s.e.m (*n* = 4). ANOVA: *, **, and *** indicate significant differences at *p* < 0.05, *p* < 0.01, and *p* < 0.001. PUE = phosphorus (P) use efficiency.

The genotype × P interaction significantly affected shoot P concentration, root P content, seed P content, PUE, and physiological PUE (*p* < 0.05, *p* < 0.01, *p* < 0.001, Supplementary Table S3, Figure 8a–d). PI 595362 had a significantly greater shoot P concentration than PI 561271 under P120 but not other P levels (Supplementary Table S3). In contrast, PI 561271 had 222% more root P content and 78% more seed P content than PI 595362 under P120 but not other P levels (*p* < 0.01, Figure 8c,d). While no genotype × P interactions occurred for root P concentration, seed P concentration, or shoot P content among the tested genotypes, significant differences occurred between genotypes and P levels (*p* < 0.05, Supplementary Table S3). The genotype × P interaction significantly decreased PUE and increased physiological PUE in both genotypes under P60 and P120 relative to P0. PI 561271 had the highest PUE and physiological PUE under P60 and P120 (*p* < 0.05, Figure 8a,b). Phosphorus application significantly increased root and shoot P concentrations and contents in both genotypes under P60 and P120 relative to P0.

#### 2.2.5. Yield and Yield Components of Soybean at Maturity

PI 561271 had more shoot (53%) and root (165%) dry weight than PI 595362 under P60 and P120 but no difference under P0 (*p* < 0.01 and *p* < 0.001, Table 4). Seed yield per plant had a significant genotype × P interaction effect (*p* < 0.05, Table 4), with PI 561271 producing 47% more seed yield than PI 595362 at both P60 and P120 but no differences under P0. No significant genotype or genotype × P effects occurred for pod number per plant, seed number per pod, seed number per plant, or harvest index (%), and no genotype × P interaction occurred for 100-seed weight (Table 4). However, P application increased pod and seed numbers per plant, seeds per pod, 100-seed weight, and harvest index (%), more so under P60 and P120 than P0 (*p* > 0.05, Table 4). PI 561271 produced more shoot dry weight, root dry weight, and seed yield under P60 and P120 than PI 595362 (Table 4). In addition, shoot and root dry weights and seed yield differed between genotypes under P0 (Table 4).

## 3. Discussion

### 3.1. Plant Growth, Biomass Distribution, and Root Morphological Responses to Variable P Supply

Differences in plant growth, shoot and root dry weights, and root morphological traits among soybean genotypes under different P levels indicate genotypic variability. At the vegetative stage (Experiment 1), the deep-rooted genotype PI 398595 had the highest shoot dry weight under P60 but no difference under P0 compared to other genotypes. In addition, PI 597387, PI 647960, PI 398595, and PI 561271 had more root dry weight than PI 654356 and PI 595362 under P60 but no differences at P0. Moreover, the shallow-rooted genotype PI 597387 produced more root length than the other genotypes at both P levels. These results suggest that short-duration shallow-rooted genotypes with shorter life cycles had faster root growth, producing more root dry weight and total root length than long-duration deep-rooted genotypes with longer life cycles and slower plant growth under different P levels. In other studies, shallow-rooted genotypes accumulated more P under different P supplies than cultivars with deeper root systems in soybean and barley [16,17]. Genotypes with shallow root systems had higher root length and dense lateral roots similar to our findings at the vegetative stage. Across soybean genotypes, P supply regulated root and shoot growth mainly through the root distribution pattern, which is essential for adapting to nutrient-deficit environments [18,19]. The long-duration, deep-rooted PI 398595 and PI 561271 had thinner roots than the other genotypes and, thus, greater P acquisition, as previously reported [20]. At the flowering stage, the deep-rooted genotype PI 561271 had greater leaf areas, shoot, root dry weights, total dry weight, and root lengths than the shallow-rooted genotype PI 595362 under P60 and P120 but no differences under P0, with similar trends found at maturity. Deep-rooted genotypes take longer to flower and mature than shallow-rooted genotypes. A recent study suggested that the tap-rooted legumes lupin, soybean, faba bean, and chickpea had greater root growth responses to P supply than the fibrous-rooted species maize, wheat, and canola [12]. The root:shoot ratio of legume species did not significantly vary in response to P application [12], consistent with our findings. Several studies have shown that applying P to soil enhances soybean growth and dry matter [10,21]. In this study, soybean growth responses to P supply varied among genotypes with contrasting root system sizes and variations in life cycles. Phosphorus supply increased the biomass of all tested genotypes, with root morphological traits consistent with other studies [12,22].

### 3.2. Root Physiological Responses under Different P Supply

Rhizosphere carboxylase activity and PUE are essential for adaptation to a stressful environment mainly through a genotypic variation in root distribution and development pattern [18,19]. Root exudation of carboxylates is necessary for P to mobilize into the soil and become more available for plant uptake and acquisition [23]. Our study showed that the proportion of carboxylates and total carboxylates varied due to genotypic differences, variation of growth cycles, and root system architectural patterns under different P supplies. At the vegetative stage, PI 654356 produced significantly more total carboxylates than PI 647960 under P60 but no differences under P0. In addition, PI 654356 secreted significantly more citrate than PI 597387 under P60. At the flowering stage, PI595362 had a greater proportion of carboxylates as malonate and malate and more total carboxylates than PI 561271 under P60 and P120 but did not differ under P0. Malate, malonate, and citrate were the main rhizosphere carboxylates in the six soybean genotypes at the vegetative and flowering stages. Like other legume species in response to P deficiency [24], citrate and malate are the two most important organic acids by the role for symbiotic nitrogen fixation, phosphorus acquisition, and aluminum tolerance.

Moreover, oxalate and malate were the prime organic acids identified in different studies of soybean [25,26]. In another study, the root exudates of P-efficient and P-adequate soybean plants mainly comprised malonate [27]. The P applications in this study stimulated carboxylate exudation in soybean genotypes. Similarly, some grain legumes, including soybean, increased the exudation of total carboxylates into the rhizosphere in response to increasing P dose [28,29], with similar results reported by [12] for chickpea, maize, and wheat. Under P-deficient conditions, soybean roots exuded more carboxylates than maize but much less than chickpea, faba bean, and lupin [30]. In other studies, alfalfa, lupin, and chickpea increased carboxylate exudation into the rhizosphere in response to P deficiency [7,31,32]. Thus, carboxylates are essential for mobilizing inorganic P in the rhizosphere and P uptake by plant roots, as indicated by positive correlations between total carboxylates and shoot P content, root P content, and physiological PUE in soybean reported in this study and elsewhere [33], narrow-leafed lupin [34], chickpea [7], and brassica [35].

All soybean genotypes significantly increased root, shoot, and seed P concentrations, contents, and physiological PUE in the P-added treatments relative to P0 at different growth stages. At the vegetative stage, PI 398595, PI 647960, and PI 561271 had 54% more root P content than PI 654356, PI 595362, and 597387 under P60 but no differences under P0. The deep-rooted genotypes PI 398595, PI 647960, PI 654356, and PI 561271 had higher PUE than the shallow-rooted genotypes PI 595362 and PI 597387. In most grain legumes, including soybean and lupin, P concentrations and contents increased significantly with increasing P levels [10,28,36], consistent with the present findings. Deep-rooted genotypes at the flowering stage had greater shoot and root P contents than shallow-rooted genotypes under P120 but did not differ at P60 and P0. Moreover, PI 561271 had greater root P content, seed P content, PUE, and physiological PUE than PI 595362 under P60 and P120 but no differences under P0, but PI 595362 had the highest shoot P concentration during later growth and at physiological maturity. Similarly, P-efficient wheat lines had higher shoot P contents and root growth than inefficient lines [37]. The high shoot and root dry weights and P concentrations in soybean likely increased total root length and decreased root diameter, increasing shoot and root P concentrations and contents and PUE [10]. In the present study, at different growth stages, agronomic PUE decreased, and physiological PUE increased with increasing P addition. Increasing application of phosphate fertilizer decreased agronomic efficiency in pasture legumes [38] and increased physiological PUE in chickpeas [6] and soybean [39]. The current study indicated that root size is necessary for P acquisition, with a strong correlation between shoot P content and root dry weight, consistent with other studies on narrow-leafed lupin [34] and chickpea [6]. Thus, P application significantly increased shoot and root P concentrations, shoot and root P contents, and PUE at the vegetative stage in the long-duration, deep-rooted genotypes relative to the short-duration, shallow-rooted genotypes, with similar trends at the reproductive and maturity stages. Overall, the short-duration, shallow-rooted genotypes exuded more total carboxylates than the long-duration, deep-rooted genotypes, while the opposite was true for PUE under different P supplies.

### 3.3. Yield and Yield-Contributing Traits under Different P Supply

Increasing P availability to meet crop requirements enables consistent biomass and grain yield production under reduced fertilizer addition. In this study, PI 561271 and PI 595362 responded best to P fertilizer application with the highest dry matter and seed yield production. The study indicated that soybean seed yield is directly related to plant P accumulation rate. A recent study showed that seed yield positively correlated with P accumulation in different plant parts under various P supplies [40]. The current study showed that without external P supply, it significantly reduced shoot and root dry weights and seed yield, consistent with previous studies on soybean [13,41]. The minor decrease in seed yield under P deficiency in P-efficient soybean than in P-inefficient may be evolved by pod number per plant [33].

## 4. Material and Methods

### 4.1. Plant Material and Growth Conditions (Experiment 1)

This study used six contrasting soybean genotypes (PI 597387, PI 647960, PI 398595, PI 561271, PI 654356, and PI 595362) based on their contrasting root morphological traits at the seedling (semi-hydroponic system) and flowering stage (1.0 m large soil-filled rhizoboxes) when grown under glasshouse conditions [42]. Plants were grown in rectangular plastic pots filled with 1.3 kg of soil mixed with coarse river sand in a ratio of 3:1 by weight (W:W). The soil was collected from the University of Western Australia (UWA) Future Farm, Pingelly (32°300′ S, 116°590′ E). A transparent polyethylene sleeve (12 cm × 12 cm, 105 µm thick) without a hole was placed inside each pot for ease of harvest and to control nutrient loss. Single superphosphate was used as the phosphorus (P) source for two P treatments; low P (P0) received no P, and added P (P60) received 60 mg P kg^−1^ dry soil [11]. The experiment was completed in a controlled glasshouse from October 2021 to November 2021. The average daily air temperature was around 22 °C (max/min 28/16 °C), with 64% relative humidity and 11–12 h natural light. A two-factorial (six contrasting genotypes and two P levels) experiment with four replicates (48 plastic pots) was conducted followed by a randomized complete block design. At 49 days after sowing (DAS), all pots were harvested for initial shoot and root growth, PUE, and root exudation measurements.

### 4.2. Plant Material and Growth Conditions (Experiment 2)

Two contrasting soybean genotypes (PI 561271 and PI 595362) selected from our previous studies [42] and Experiment 1 were used. Plants were grown in polyvinyl chloride cylindrical (PVC) columns (100 cm depth and 9 cm diameter). A long, transparent polyethylene sleeve (110 cm × 15 cm, 105 µm thick) without a hole was placed inside each PVC column for easy harvest and to control nutrient loss. Each PVC column was filled with 9.0 kg of sandy loam soil and coarse river sand mixture (3:1 ratio by weight). The experiment had three P treatments, using single superphosphate as the P source: low P (P0) received no P, medium P (P60) received 60 mg P kg^−1^ in dry soil, and high P (P120) received 120 mg P kg^−1^ in dry soil [11]. This experiment was completed from October 2021 to March 2022 in the same controlled glasshouse as Experiment 1. The experiment had a randomized complete block design with two factors (two genotypes and three P levels), two harvests, and four replicates (48 PVC columns). Half of the PVC columns (12 per genotype) were harvested at the appearance of the first flower [49 DAS (PI 595362) and 67 DAS (PI 561271)] to record initial shoot and root traits, PUE, and carboxylase exudation. The remaining columns were harvested at maturity when 95% of the pods had turned brown [43] [105 DAS (PI 595362) and 143 DAS (PI 561271)] to record shoot and root traits, PUE, yield, and yield-contributing traits.

### 4.3. Soil Preparation, Fertilizer, and Water Application (Both Experiments)

CSBP Soil and Plant Laboratory (Bibra Lake, WA, Australia) analyzed soil samples for physical and chemical properties. The soil’s physical and chemical properties were listed in Table 5. Basal nutrients [190 mg N kg^−1^ dry soil and 130 mg K kg^−1^ dry soil [44]] were applied to the whole soil (Experiment 1) and equally distributed in the top 20 cm soil layers in each PVC column (Experiment 2) to mimic field fertilizer application. Urea and potash sulfur was used as N and K sources. In addition, gypsum and elemental sulfur were applied to maintain the same nutrients in all experimental units. After emergence, plants were thinned to two plants per plastic pot or one per PVC column. Both experiments followed the same procedures for sterilizing soybean seeds, rhizobial inoculation, fertilization, and water application as described elsewhere [42].

### 4.4. Above- and Below-Ground Traits Measurement (Both Experiments)

Destructive above- and below-ground measurements occurred at 49 DAS (Experiment 1) and at the time of appearance of the first flower and plant maturity (Experiment 2). Rhizosphere pH, shoot P concentration, and chlorophyll content were measured. Chlorophyll content was measured by using SPAD 502 plus meter at between 10.0 and 12.0 day time [45]. Immediately after harvesting the shoots, the plastic sleeve was carefully pulled out of each PVC column, and the plastic pot and cut longitudinally. The root system was recovered from the soil before collecting the rhizosphere root exudation. After root exudation collection, recovered roots were placed in plastic bags and stored in a 4 °C cold room until root scanning following the standard procedure and morphological trait analysis using WinRhizo Pro software (v2009, Regent Instrument, Quebec, QC, Canada) described in [42].

### 4.5. Root Exudation Collection and Measurements (Both Experiments)

The root exudation procedure followed an established method routinely used in our laboratory [46]. The HPLC vials were used for the collection of samples, placed on dry ice during the extraction procedures, and stored at −20 °C until the carboxylates analysis was performed using HPLC [47], except for oxalate, which was determined using the method described earlier [48]. Total carboxylates are the sum of those detected in the root rhizosphere.

### 4.6. Shoot and Root P Acquisition and PUE (Both Experiments)

Approximately 20 mg subsamples of finely ground shoots (stems and leaves) and roots (49 DAS and appearance of first flower) and seeds (maturity) were added to hot concentrated nitric and perchloric acids for acid digestion (3:1, v:v) as detailed elsewhere (Pang et al., 2010). The samples were ground to a fine powder using a coffee grinder. Shoot, root, and seed P concentrations were measured using a UV–VIS spectrophotometer followed by the malachite green method [49]. Shoot and root P content was obtained from the P concentration and the corresponding dry weight. Physiological P use efficiency was determined as the ratio of shoot dry weight to shoot P concentration [50]. Agronomic PUE was determined as the increase in shoot yield per unit of P fertilizer supply [38,50].

### 4.7. Yield and Yield-Contributing Traits of Soybean at Maturity (Experiment 2)

At maturity, [105 DAS (PI 595362) and 143 DAS (PI 561271)], the plants were cut at ground level. Total pod and seed numbers and seed weight were recorded. The harvest index was measured as the seed weight ratio to total above-ground dry weight, and other parameters related to yield were determined as described in [42].

### 4.8. Statistical Analyses

XLSTAT (2021.2.2 version) was used for data analysis, including analysis of variance (ANOVA). Two-way ANOVA was measured at 49 DAS (Experiment 1) and flowering and maturity (Experiment 2). Tukey’s honest significance difference (HSD) test was used to compare genotype, P, and genotype × P effects (both experiments). Pearson’s correlation and principal component analysis by using R were performed separately on each vital parameter from Experiment 1. The two-way genotype × P was calculated when significant at *p* < 0.05; otherwise, significant main effects (genotypes or P) are presented.

## 5. Conclusions

Soybean genotypes supplied with P regardless of rate of application had the highest P use efficiency, root exudation, root dry weight, total root length, shoot dry weight, and yield and yield-contributing traits relative to those without P, which could increase energy storage in the P-amended plant. Moreover, applied P nutrition could also enhance P use efficiency of soybean genotypes which helps for sustainable soybean production around the globe. Future studies are needed on drought resistance in soybean by improving the growth of roots by P application to extract water from deeper soil layers or improving water conservation in the plant’s tissues in water-shortage environments.

## Figures and Tables

**Table 1:** Mean data of root and shoot traits of six soybean genotypes grown in rectangular pots (Expt. 1). Values are the mean of four replicates (1 plant pot^−1^). The plants were harvested 49 days after sowing (DAS) under 0 (P0) and 60 (P60) mg kg^−1^ dry soil. Mean data of each trait followed by different letters differ significantly at *p* < 0.05 using Turkey’s test.

Genotypes	P Rates	Shoot Dry Weight	Root Dry Weight	Total Dry Weight	Root:Shoot Ratio	Average Root Diameter	Total Root Length
		(g plant^−1^)	(g plant^−1^)	(g plant^−1^)		(mm)	(m)
PI 561271	P0 P60	0.71c 2.55b	0.26e 0.83ab	0.97de 3.38bc	0.36cde 0.33cde	0.43 0.45	21.4h 67.1bcd
PI 398595	P0 P60	1.15c 3.39a	0.60c 1.03a	1.75d 4.42a	0.53a 0.31de	0.40 0.42	50.3def 85.2b
PI 654356	P0 P60	0.58c 2.42b	0.25e 0.65bc	0.84e 3.07bc	0.43abcd 0.27e	0.46 0.48	18.8h 46.7efg
PI 647960	P0 P60	0.77c 2.63b	0.36de 1.01a	1.14de 3.63ab	0.47abc 0.38bcde	0.46 0.48	30.5gh 81.9b
PI 595362	P0 P60	0.75c 2.04b	0.34de 0.72bc	1.09de 2.76c	0.46abc 0.37bcde	0.44 0.46	35.9fgh 73.6bc
PI 597387	P0 P60	1.11c 2.63b	0.56cd 1.00a	1.67de 3.63ab	0.51ab 0.38bcde	0.44 0.46	61.0cde 106.0a
	Genotype	***	***	***	***	***	***
	P rate	***	***	***	***	***	***
	Genotype × P	*	*	*	*	ns	*

ANOVA: ns. = non-significant, * and *** indicate significant difference at *p* < 0.05 and *p* < 0.001.

**Table 2:** Pearson’s correlation matrix of root morphological and physiological traits in the soybean genotypes from Experiment 1. The plants were harvested 49 days after sowing (DAS) under 0 (P0) and 60 (P60) mg kg^−1^ dry soil.

Measurable Traits	Shoot Dry Weight	Root Dry Weight	Total Root Length	OXALIC ACID	Malic Acid	Malonic Acid	Citric Acid	Total Carboxylates	Shoot P Content	Root P Content
Root dry weight	0.90 ***									
Total root length	0.81 ***	0.95 ***								
Oxalic acid	0.55 ***	0.49 ***	0.53 ***							
Malic acid	0.89 ***	0.75 ***	0.61 ***	0.51 ***						
Malonic acid	0.82 ***	0.79 ***	0.79 ***	0.62 ***	0.76 ***					
Citric acid	0.60 ***	0.45 ***	0.29 *	0.35 **	0.76 ***	0.33 *				
Total carboxylates	0.91 ***	0.79 ***	0.69 ***	0.62 ***	0.97 ***	0.84 ***	0.75 ***			
Shoot P content	0.93 ***	0.81 ***	0.74 ***	0.60 ***	0.86 ***	0.82 ***	0.52 ***	0.87 ***		
Root P content	0.91 ***	0.88 ***	0.78 ***	0.48 ***	0.84 ***	0.72 ***	0.53 ***	0.83 ***	0.88 ***	
Physiological P-use efficiency	0.77 ***	0.76 ***	0.68 ***	0.22ns	0.61 ***	0.54 ***	0.50 ***	0.63 ***	0.51 ***	0.64 ***

ANOVA: ns. = non-significant, *, **, and *** indicate significant differences at *p* < 0.05, *p* < 0.01, and *p* < 0.001.

**Table 3:** Mean root and shoot trait data for two soybean genotypes grown in 1.0 m PVC columns (Expt. 2). Values are mean of four replicates (1 plant column^−1^). The plants were harvested at the flowering stage (appearance of first flower) at 49 DAS (PI 595362) and 69 DAS (PI 561271) under 0 (P0), 60 (P60), and 120 (P120) mg kg^−1^ dry soil.

Genotypes	P Rate	Shoot Dry Weight	Root Dry Weight	Total Dry Weight	Root:Shoot Ratio	Average Root Diameter	Total Root Length
		(g plant^−1^)	(g plant^−1^)	(g plant^−1^)		(mm)	(m)
PI 561271	P0 P60 P120	0.79d 7.74b 10.25a	0.52d 4.24b 5.40a	1.31d 11.97b 15.65a	0.65 0.55 0.54	0.41 0.44 0.45	46.1e 327.9b 440.3a
PI 595362	P0 P60 P120	0.80d 3.75c 4.74c	0.36d 1.64c 2.32c	1.16d 5.39c 7.06c	0.45 0.45 0.50	0.42 0.44 0.46	41.4e 175.8d 244.1c
	Genotype	***	***	***	**	ns	***
	P rate	***	***	***	ns	*	***
	Genotype × P rate	***	***	***	ns	ns	***

Mean data for each trait followed by different letters differ significantly at *p* < 0.05 using Turkey’s test. ANOVA: ns. = non-significant, *, **, and *** indicate significant difference at *p* < 0.05, *p* < 0.01, and *p* < 0.001.

**Table 4:** Yield and yield contributing traits of two soybean genotypes grown in 1.0 m-deep PVC columns (Expt. 2). Values are four replicates (1 plant column^−1^). The plants were harvested at maturity at 105 DAS (PI 595362) and 143 DAS (PI 561271) under 0 (P0), 60 (P60), and 120 (P120) mg kg^−1^ dry soil.

Genotypes	P Rates	Shoot Dry Weight	Root Dry Weight	Pods/ Plant	Seeds/Pod	Seeds/ Plant	100 Seeds Weight	Seed Yield/Plant	Harvest Index (%)
		(g)	(g)				(g)	(g)	
PI 561271	P0	1.61c	0.81cd	5	2	10	15.10	1.51c	40b
	P60	12.33ab	4.72a	22	3	66	15.30	10.10a	44ab
	P120	15.70a	5.91a	25	3	75	16.00	12.00a	48a
PI 595362	P0	1.06c	0.50d	4	2	8	8.62	0.69c	41ab
	P60	9.21b	1.97bc	21	3	63	11.41	7.19b	44ab
	P120	9.76b	2.03b	23	3	69	11.59	8.00b	47ab
	Genotype	***	***	ns	ns	ns	***	***	ns
	P rate	***	***	***	***	***	*	***	**
	Genotype × P rate	**	***	ns	ns	ns	ns	*	ns

Mean data for each trait followed by different letters differ significantly at *p* < 0.05 using Turkey’s test. ANOVA: ns. = non-significant, *, **, and *** indicate significant differences at *p* < 0.05, *p* < 0.01, and *p* < 0.001.

**Table 5:** Soil’s chemical properties before being mixed with sand.

Property	Value
Texture	2
Ammonium nitrogen (mg kg^−1^)	<1
Nitrate nitrogen (mg kg^−1^)	4.0
Phosphorus Colwell (mg kg^−1^)	6.5
Potassium Colwell (mg kg^−1^)	117
Sulfur (mg kg^−1^)	5.1
Organic carbon (%)	0.67
Conductivity (dS m^−1^)	0.04
pH (CaCl_2_)	6.3
pH (H_2_O)	7.35
Phosphorus buffering index (PBI)	61.45

## Data Availability

Data will be made available on request.

## Supplementary Materials

The following supporting information can be downloaded at: https://www.mdpi.com/article/10.3390/plants12051110/s1, Table S1. Mean shoot and root trait data of six soybean genotypes grown in rectangular pots (Expt. 1). Values mean four replicates (1 plant pot^−1^). The plants were harvested 49 days after sowing (DAS) under 0 (P0) and 60 (P60) mg kg^−1^ dry soil. Mean data for each trait followed by different letters differ significantly at *p* < 0.05 using Tukey’s *t*-test. ANOVA: ns. = non-significant, * and *** indicate significant differences at *p* < 0.05 and *p* < 0.001. Table S2. Mean shoot and root trait data for two soybean genotypes grown in 1.0 m deep PVC columns (Expt. 2). Values are mean of four replicates (1 plant column^−1^). The plants were harvested at the flowering stage (appearance of the first flower) at 49 DAS (PI 595362 (49 DAS) and 67 DAS (PI 561271) under 0 (P0), 60 (P60) and 120 (P120) mg kg^−1^ dry soil. Mean data for each trait followed by different letters differ significantly at *p* < 0.05 using Turkey’s test. ANOVA: ns. = non-significant, *, ** and *** indicate significant differences at *p* < 0.05, *p* < 0.01 and *p* < 0.001. Table S3. Mean data for different phosphorus (P) concentrations and content of two soybean genotypes grown in 1.0 m deep PVC columns (Expt. 2). Values are means of four replicates (1 plant column^−1^). The plants were harvested at maturity at 105 DAS (PI 595362) and 143 DAS (PI 561271 ) under 0 (P0), 60 (P60) and 120 (P120) mg kg^−1^ dry soil. Mean data of each trait followed by different letters differ significantly at *p* < 0.05 using Turkey’s test. ANOVA: ns. = non-significant, *, ** and *** indicate significant difference at *p* < 0.05, *p* < 0.01 and *p* < 0.001.
