# Supplementary material for: Phosphorus Application Enhances Root Traits, Root Exudation, Phosphorus Use Efficiency, and Seed Yield of Soybean Genotypes
Source: Plants (Basel). 2023 Mar 1;12(5):1110. doi: 10.3390/plants12051110 (PMC10005312; doi:10.3390/plants12051110)
Supplement: Supplementary file 1 [file plants-12-01110-s001.zip › plants-2201659-supplementary.pdf]

Supplementary Table S1. Mean shoot and root trait data of six soybean genotypes grown in rectangular pots (Expt. 1). Values mean four replicates (1 plant pot $^{-1}$). The plants were harvested 49 days after sowing (DAS) under 0 (P0) and 60 (P60) mg kg^{-1} dry soil. Mean data for each trait followed by different letters differ significantly at *P* < 0.05 using Tukey's t-test. ANOVA: ns. = non-significant, * and *** indicate significant differences at *P* < 0.05 and *P* < 0.001.

Genotypes	P rates	Plant height	SPAD	Leaf area	Root length density
		(cm)		(cm^2 plant $^{-1}$)	(cm cm3)
PI 561271	P0	24.75d	31.50d	145gh	1.64h
	P60	28.50bcd	42.12b	527ab	5.16bcd
PI 398595	P0	32.25abcd	31.15d	221fg	3.87def
	P60	35.75ab	40.87b	614a	6.55b
PI 654356	P0	24.75d	31.42d	105h	1.45h
	P60	29.00bcd	36.67c	330de	3.59efg
PI 647960	P0	31.25abcd	37.20c	115h	2.34gh
	P60	39.75a	42.17b	360cd	6.30b
PI 595362	P0	23.75d	35.12c	105h	2.76fgh
	P60	35.25abc	44.95a	250ef	5.66bc
PI 597387	P0	27cd	32.07d	160fgh	4.69cde
	P60	39.75a	40.82b	453bc	8.16a
ANOVA	Genotype	***	***	***	***
	P rates	***	***	***	***
	Genotype × P rates	*	***	***	*

Supplementary Table S2. Mean shoot and root trait data for two soybean genotypes grown in 1.0 m deep PVC columns (Expt. 2). Values are mean of four replicates (1 plant column $^{-1}$). The plants were harvested at the flowering stage (appearance of the first flower) at 49 DAS (PI 595362 (49 DAS) and 67 DAS (PI 561271) under 0 (P0), 60 (P60) and 120 (P120) mg kg^{-1} dry soil. Mean data for each trait followed by different letters differ significantly at $P < 0.05$ using Turkey's test. ANOVA: ns. = non-significant,*, **and *** indicate significant differences at $P <$ 0.05, $P < 0.01$ and $P < 0.001$.

Genotypes	P rates	Shoot P concentration	Root P concentration	Days to first flower	Leaf area	Plant height	Root length density	Root surface area	SPAD	Rhizosphe re pH	Root volume
		(mg g^{-1} DW)	(mg g^{-1} DW)		(cm^2 plant $^{-1}$)	(cm)	(cm cm^3)	(cm^2)			(cm^3)
PI 561271	P0	1.33c	1.32d	67a	202d	21.00c	0.59e	194.00e	28.77	6.40a	4.46d
	P60	2.89b	2.72cd	67a	1387b	36.50a	4.18b	723.75b	41.77	5.98bcd	14.79bc
	P120	5.28a	5.45b	64a	2019a	36.25a	5.61a	1141.50a	41.37	5.69d	23.93a
PI 595362	P0	1.08c	1.50d	49b	112d	19.25c	0.53e	183.50e	29.50	6.28ab	3.77d
	P60	2.66b	3.41c	43c	491c	30.00b	2.24d	357.25d	43.42	6.13abc	12.57c
	P120	6.51a	6.91a	41c	635c	33.75ab	3.11c	533.25c	42.60	5.82cd	17.28b
	Genotype	*	**	***	***	***	***	***	ns	*	***
	P rate	***	***	***	***	***	***	***	***	***	***
	Genotype × P	**	*	*	***	*	***	***	ns	*	***

Supplementary Table S3. Mean data for different phosphorus (P) concentrations and content of two soybean genotypes grown in 1.0 m deep PVC columns (Expt. 2). Values are means of four replicates (1 plant column $^{-1}$). The plants were harvested at maturity at 105 DAS (PI 595362) and 143 DAS (PI 561271) under 0 (P0), 60 (P60) and 120 (P120) mg kg^{-1} dry soil. Mean data of each trait followed by different letters differ significantly at *P*< 0.05 using Turkey's test. ANOVA: ns. = non-significant, *, ** and *** indicate significant difference at *P*< 0.05, *P*< 0.01 and *P*< 0.001.

Genotypes	P rates	Shoot P concentration	Root P concentration	Seed P concentration	Shoot P content
		(mg g^{-1} DW)	(mg g^{-1} DW)	(mg g^{-1} DW)	(mg plant^{-1})
PI 561271	P0	0.79d	0.87	4.06	1.26
	P60	1.48cd	1.34	5.50	18.22
	P120	1.90c	2.30	6.50	29.75
PI 595362	P0	2.17bc	0.74	3.80	2.33
	P60	2.83b	1.11	4.95	25.88
	P120	4.17a	2.08	5.53	40.96
	Genotype	***	*	***	***
	P rate	***	***	***	***
	Genotype × P	**	ns	ns	ns
